# Association between the C-reactive protein–albumin–lymphocyte (CALLY) index and osteoarthritis prevalence: a cross-sectional study of American adults based on NHANES

**DOI:** 10.3389/fnut.2025.1710682

**Published:** 2025-12-15

**Authors:** Linzeng Qi, Qian Chen, Yingxia Li, Yongyuan Guo

**Affiliations:** Department of Orthopaedics, Qilu Hospital of Shandong University, Jinan, China

**Keywords:** osteoarthritis, prevalence, nutrition, inflammation, immunity, body mass index, CALLY index

## Abstract

**Background:**

Osteoarthritis (OA) is a common degenerative joint disease, and accumulating evidence suggests that metabolic and inflammatory pathways are involved in its pathogenesis. The C-reactive protein–albumin–lymphocyte (CALLY) index, an integrated biomarker incorporating serum albumin, lymphocyte counts, and C-reactive protein (CRP), has recently attracted attention as a potential indicator of systemic inflammation and nutritional status. However, its association with OA prevalence remains unclear.

**Methods:**

We conducted a cross-sectional analysis using data from the National Health and Nutrition Examination Survey (NHANES) cycles 2001–2010 and 2015–2018. To assess external consistency, we additionally analyzed an independent hospital-based cohort (n = 1,534) and evaluated the association between the CALLY index and OA prevalence using multivariable logistic regression (weighted in NHANES), restricted cubic spline (RCS) modeling, interaction tests, and subgroup analyses. Because the CALLY index exhibited a right-skewed distribution, we conducted a pre-specified sensitivity analysis using its natural logarithm ln(CALLY). All models were adjusted for key covariates, including age, educational attainment, and additional potential confounders.

**Results:**

The untransformed CALLY index exhibited marked right skewness, which was substantially reduced after log transformation. In the NHANES analyses, higher levels of both the raw CALLY index and ln(CALLY) were significantly associated with a lower prevalence of OA. Restricted cubic spline analyses revealed a nonlinear inverse association on the raw scale and an approximately linear inverse association on the ln(CALLY) scale. In the external validation cohort, the direction of the associations was consistent.

**Conclusion:**

In both the NHANES-based cross-sectional analyses and the external validation cohort, the CALLY index was inversely associated with OA prevalence. On the raw scale, this association demonstrated a nonlinear pattern, whereas on the log-transformed scale ln(CALLY), it was approximately linear. These findings remained robust to extensive covariate adjustment and multiple sensitivity analyses.

## 1 Introduction

Osteoarthritis (OA) is the most common chronic, degenerative joint disorder worldwide. In the context of global population aging, the incidence of OA has steadily increased, rising by 132.2% between 1990 and 2020, and is projected to increase by a further 60–100% by 2050 (1). As a result, OA has become a major public health concern, particularly among middle-aged and older adults (2, 3). Clinically, OA is characterized by joint pain, morning stiffness, and crepitus, which may progress to joint instability and physical disability, severely impairing quality of life and contributing to escalating healthcare costs. Accordingly, early identification and standardized management of OA are believed to help reduce the disease burden among individuals with, or at risk of, advanced-stage disease.

The pathogenesis of OA is multifactorial, involving a complex interplay of biomechanical and biological factors such as sex, age, obesity, chronic low-grade inflammation, nutritional and metabolic imbalances, genetic susceptibility, and previous joint injuries (4–9). Emerging evidence highlights the critical role of excessive mechanical loading in the initiation and progression of OA. Excessive mechanical stress disrupts chondrocyte homeostasis and can induce ferroptosis via Piezo1-mediated calcium influx, leading to downregulation of glutathione peroxidase 4 (GPX4) and consequent mitochondrial oxidative damage (10). In parallel, mechanical overload has been shown to upregulate reticulocalbin 2 (Rcn2), thereby exacerbating inflammation and extracellular matrix degradation in articular cartilage (11).

Recent studies have also demonstrated the biomechanical significance of the infrapatellar fat pad (IFP) and suprapatellar fat pad (SFP) in knee OA progression. These fat pads are not merely space-filling tissues; they are increasingly recognized as active modulators of joint biomechanics and local inflammatory responses. The OA-associated IFP exhibits markedly increased stiffness compared with the SFP, suggesting pathological remodeling that may alter intra-articular load distribution and promote cartilage degeneration. Although the SFP is mechanically less stiff, its anatomical location enables it to influence patellofemoral alignment during knee extension, potentially contributing to joint mechanical stability under pathological conditions (12).

The OA is increasingly recognized as a whole-joint disease involving multiple articular structures, including the cartilage, synovium, menisci, tendons, ligaments, and periarticular adipose tissue. These components actively contribute to disease pathogenesis rather than merely undergoing passive degeneration. For instance, chondrification and degenerative changes in the anterior cruciate ligament (ACL) and meniscotibial ligament can alter joint mechanics and impair stability, thereby accelerating OA progression (13). Similarly, meniscal degeneration is a key pathological event that exacerbates cartilage degeneration, playing a central role in the onset of OA (14). During disease progression, chondrocytes release various inflammatory and catabolic mediators—such as cytokines, chemokines, and adipokines—via paracrine signaling, thereby perpetuating extracellular matrix breakdown and joint inflammation. Notably, chondrocytes from OA patients release small extracellular vesicles (sEVs) enriched with connexin 43 (Cx43) that induce cellular senescence in neighboring chondrocytes, synoviocytes, and osteoblasts, thereby fostering a chronically degenerative intra-articular environment (15).

Given this pathophysiological complexity, identifying modifiable risk factors for OA is crucial to the development of effective preventive and therapeutic strategies. Recent evidence has indicated that individuals with asthma or atopic dermatitis are at increased risk of OA, potentially mediated by mast cell activation and associated synovitis (16). Furthermore, differences in circulating biomarkers such as vitamin C, vitamin K, and interleukins between patients with OA and healthy controls have been reported, suggesting their potential application in OA risk prediction (17, 18). However, many of these biomarkers are either costly or difficult to measure in routine clinical settings, which limits their practical utility. Therefore, there is a pressing need for accessible, cost-effective biomarkers that capture both systemic inflammatory burden and nutritional status for the risk stratification of OA.

The C-reactive protein–albumin–lymphocyte (CALLY) index is a composite biomarker that integrates serum albumin, lymphocyte counts, and C-reactive protein (CRP) levels to reflect both systemic inflammatory burden and nutritional status (19). It has been shown to predict overall survival in patients with colorectal cancer (CRC) more accurately than traditional prognostic indices (20). Beyond oncology, the CALLY index has also demonstrated predictive value in various chronic conditions, including cardiovascular disease, sarcopenia, and retinopathy (21). These findings support its potential as a clinically applicable biomarker in conditions associated with systemic inflammation.

Despite these advances, the association between the CALLY index and OA prevalence remains poorly characterized. Previous studies, such as that by Wang et al. (22), have employed linear regression models to examine predictors of physical function in patients with knee OA. However, linear models may fail to capture the complexity of non-linear associations inherent in OA pathophysiology. Therefore, more flexible statistical approaches—such as restricted cubic spline (RCS) modeling—are warranted to accurately characterize the association between the CALLY index and OA prevalence.

This study aimed to examine the association between the CALLY index and prevalent OA in a nationally representative sample and to assess whether patterns were consistent in an independent hospital-based cohort. We also characterized exposure–response features on the raw and ln(CALLY) scales using flexible modeling, while maintaining an association-based interpretation aligned with the cross-sectional design.

## 2 Materials and methods

### 2.1 Data source and study population

This study utilized publicly available data from the National Health and Nutrition Examination Survey (NHANES), a population-based program conducted by the National Center for Health Statistics (NCHS), a division of the Centers for Disease Control and Prevention (CDC). NHANES employs a rigorous multistage probability sampling design to assess the health and nutritional status of the non-institutionalized civilian population in the United States through structured interviews, physical examinations, and laboratory testing. The National Research Ethics Board approved all data collection protocols, and informed consent was obtained from all participants. Detailed information on the NHANES study design, sampling strategy, and data documentation is available at https://www.cdc.gov/nchs/nhanes.

**Figure 1:**
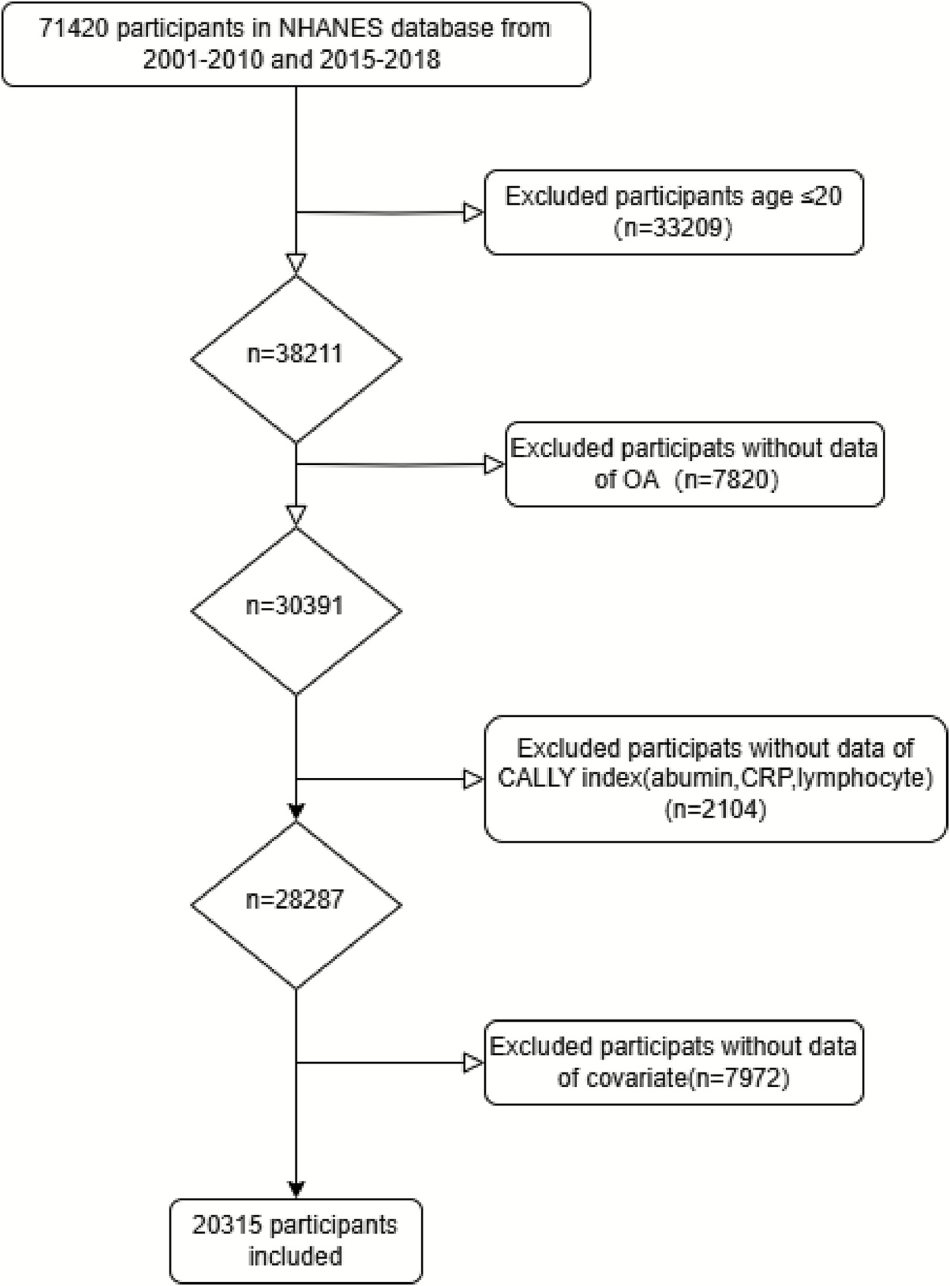
Flow chart of the study participants. Flow diagram showing the inclusion and exclusion process for participants from the NHANES database (2001–2010 and 2015–2018). A total of 71,420 participants were initially considered. After excluding individuals aged ≤20 years and those with missing data on OA status, CALLY index components (albumin, CRP, lymphocyte), and covariates, a final sample of 20,315 participants was included in the analysis.

For the present analysis, data from NHANES cycles spanning 2001–2010 and 2015–2018 were combined, covering a total of 14 years. The initial dataset included 71,420 participants. The study included potential confounders related to the CALLY index and OA, encompassing demographic characteristics and established health conditions such as hypertension, diabetes, smoking status, alcohol consumption, and body mass index (BMI). Participants were excluded based on the following criteria:(1) age under 20 years (*n* = 33,209); (2) missing data on OA status or a self-reported diagnosis of arthritis types other than osteoarthritis (*n* = 7,820); (3) missing information on any of the components required to calculate the CALLY index, including serum albumin, lymphocyte count, and C-reactive protein (CRP) (*n* = 2,104); and (4) incomplete data on relevant covariates, including age, sex, race/ethnicity, BMI, education level, smoking, alcohol consumption, hypertension, and diabetes (*n* = 7,972). After applying these exclusion criteria, a total of 20,315 participants remained for the final analysis (Figure 1).

The de-identified individual-level data used for the external consistency analysis are provided as Supplementary Dataset S1. To assess cross-cohort consistency and comparability, we conducted an external consistency analysis using a retrospective cross-sectional design, based on an independent de-identified dataset derived from routine health examinations and outpatient follow-up visits at Qilu Hospital of Shandong University between June and September 2025. The analytic sample comprised 1,534 adults (≥20 years of age), of whom 505 had OA and 1,029 did not. Inclusion criteria were as follows: (1) available OA outcome data (yes/no); (2) available measurements of all three components required to calculate the CALLY index—serum albumin, lymphocyte count, and CRP—expressed in units consistent with NHANES (albumin: g/L; lymphocytes: 10^9^/L; CRP: mg/L); and (3) available data on key covariates (age, sex, educational level, BMI, smoking status, alcohol consumption, diabetes, and hypertension). Participants with missing data for any of these variables were excluded from the analysis.

### 2.2 Assessment of OA and CALLY index

In the NHANES database, OA status was determined based on self-reported responses in the “Medical Conditions” questionnaire section. Participants were initially asked, “Has a doctor or other health professional ever told you that you have arthritis?” Those who answered “No” were classified as non-OA participants for further analysis. Participants who responded “Yes” were subsequently asked, “What type of arthritis was it?” Individuals who self-reported “osteoarthritis” were defined as OA cases. Those reporting other types of arthritis (e.g., “rheumatoid arthritis” or “psoriatic arthritis”) or who did not respond to this follow-up question were classified as non-OA participants and excluded from the study (23). Prior studies have reported an approximately 81% agreement between self-reported OA status and clinical evaluation (24).

In addition to questionnaire data, each NHANES participant underwent laboratory testing, including complete blood counts and biochemical measurements. The CALLY index was calculated to reflect both inflammatory and nutritional status using the following formula:


$$\begin{array}{l} \text{CALLY Index}=\text{Albumin}\;(g/L)\times \text{Lymphocyte count}\;\\ (10^{9}/L)÷[\mathrm{CRP}\;(\mathrm{mg}/L)\times 10]. \end{array}$$

In the external validation cohort, OA status was recorded as a binary variable (yes/no), based on physician-diagnosed OA identified from electronic medical records, outpatient documentation, or confirmation by a specialist. This definition was harmonized with the OA classification used in NHANES, allowing for direct comparisons across cohorts. The CALLY index was calculated using the same formula and measurement units as in the NHANES dataset; no unit conversions were required because serum albumin (g/L), lymphocyte counts (10^9^/L), and CRP (mg/L) were directly compatible. To define CALLY index quartiles, both datasets used population-specific quartile cutoffs (Q1–Q4). The first quartile (Q1) served as the reference category in exploratory analyses of nonlinear associations and potential curve inflection points, facilitating consistent interpretation across populations.

### 2.3 Covariates

Covariates included in this study were selected based on their established relevance to OA and their routine use in prior epidemiological research, with emphasis on variables frequently applied in related studies. A total of 10 covariates were categorized into three domains:

#### 2.3.1 Demographic characteristics

- Age (≥20 years): Participants were stratified into two age groups (≤60 years and >60 years) according to the World Health Organization (WHO) criteria.
- Gender: Male or female.
- Race/Ethnicity: Categorized as Mexican American, Non-Hispanic White, Non-Hispanic Black, Other Hispanic, and Other/Multiracial.
- Education Level: Classified as less than high school, high school or equivalent, and more than high school.

#### 2.3.2 Anthropometric measurements

- Weight: Treated as a continuous variable (in kilograms).
- BMI: Categorized into three groups: underweight/normal (<25.0 kg/m^2^), overweight (25.0–29.9 kg/m^2^), and obese (≥30.0 kg/m^2^).

#### 2.3.3 Health-related questionnaire data

- Diabetes Status: Classified as yes or no, based on self-reported physician diagnosis.
- Hypertension Status: Classified as yes or no, based on self-report or use of antihypertensive medication.
- Alcohol Consumption: Measured as the average daily intake over the past 12 months.
- Smoking Status: Defined as never smokers (fewer than 100 cigarettes smoked in a lifetime) and ever smokers (100 or more cigarettes smoked in a lifetime).

Detailed definitions, measurement protocols, and data acquisition methods for all covariates are publicly available on the NHANES website provided by the CDC: www.cdc.gov/nchs/nhanes.

In the external validation cohort, variable harmonization was performed to ensure consistency with NHANES classifications. Sex was coded as male or female, and OA status was recorded as a binary variable (yes/no). Educational attainment was aligned with NHANES categories and classified as less than 9th grade, 9–11th grade (including 12th grade with no diploma), high school graduate/GED or equivalent, some college or associate degree, or college graduate or above. BMI was modeled as a continuous variable and, for subgroup analyses and interaction tests, was additionally stratified at <25 vs. ≥25 kg/m^2^. To maintain model stability and prevent complete separation, categories with very low or zero counts in the external dataset were collapsed into adjacent categories when appropriate.

### 2.4 Statistical analysis

Following the complex, multistage probability sampling design of NHANES, all analyses were weighted using the Mobile Examination Center (MEC) examination weights (WTMEC2YR), as the components of the CALLY index—CRP, serum albumin, and lymphocyte count—were measured during laboratory assessments at the MEC. To combine data across seven 2-year NHANES cycles (2001–2010 and 2015–2018), each cycle-specific weight was divided by the number of cycles (n = 7), as recommended by NHANES analytic guidelines. All analyses accounted for sampling strata and primary sampling units (PSUs) to produce nationally representative estimates. Statistical analyses were conducted using the survey package in R software.

Continuous variables were summarized as means with standard deviations (SDs), and categorical variables were presented as frequencies and percentages. Group comparisons were performed using the Kruskal–Wallis test for continuous variables and the chi-square (*χ*^2^) test for categorical variables.

**Figure 2:**
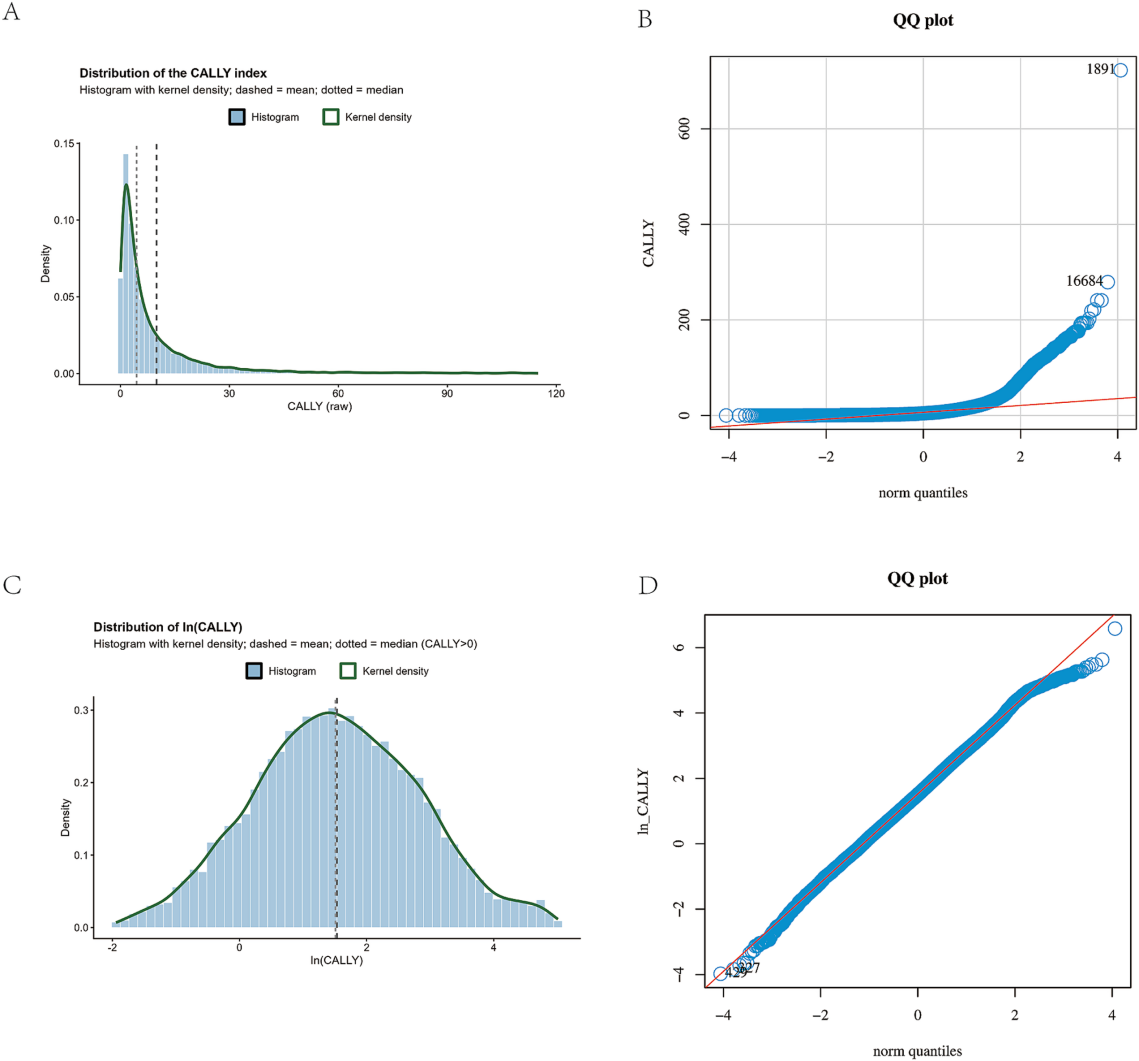
NHANES: Distribution of the CALLY Index (raw vs. log-transformed) and Normal Q–Q Plots. **(A)** Histogram of the raw CALLY index with kernel density overlay, showing marked right-skewness. **(B)** Normal Q–Q plot of raw CALLY, indicating substantial upper-tail deviation. **(C)** Histogram of ln(CALLY) (natural log), with improved symmetry and reduced skewness. **(D)** Normal Q–Q plot of ln(CALLY), showing closer alignment to normality with residual tail departures.

**Figure 3:**
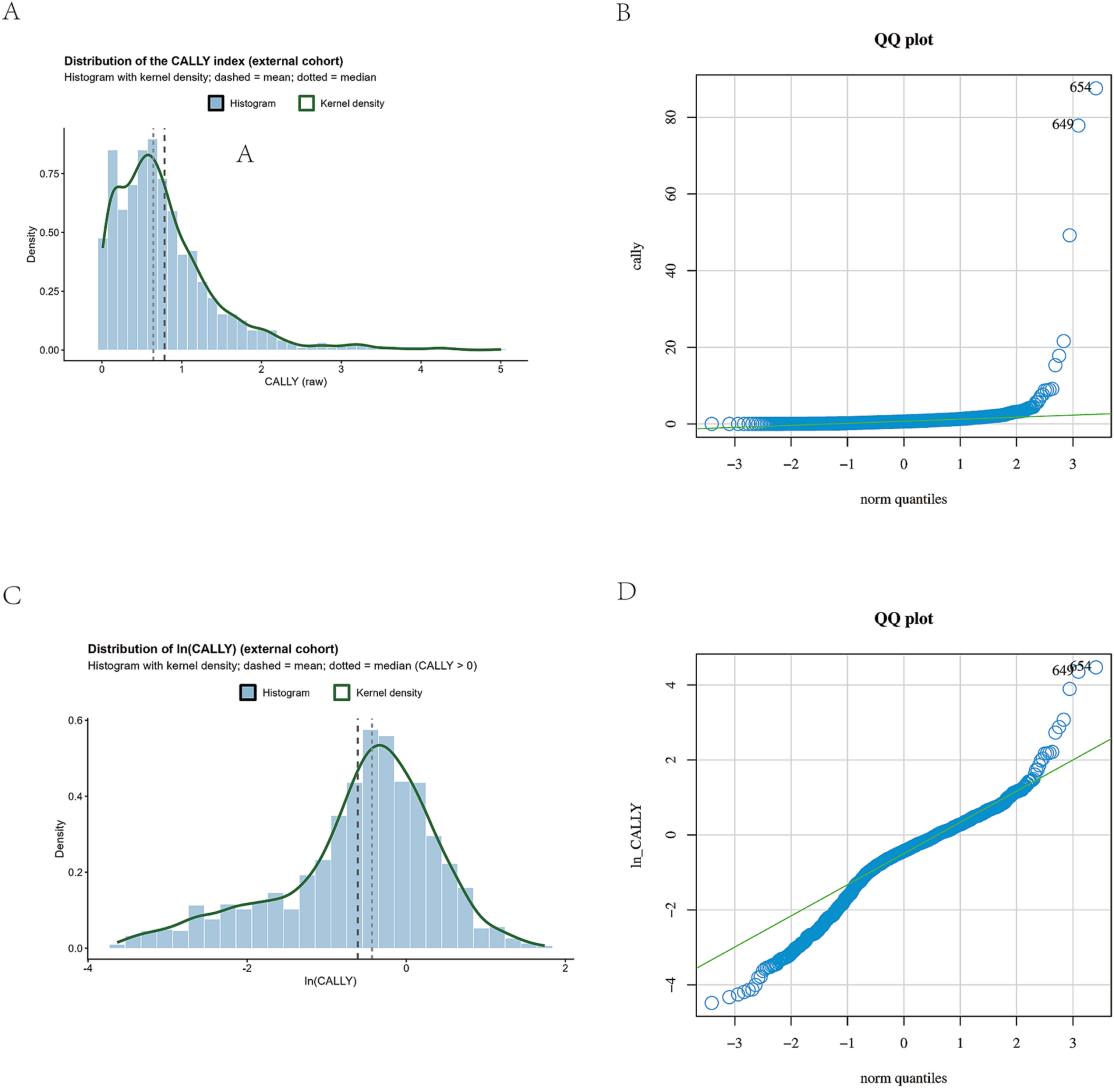
External cohort: Distribution of the CALLY index (raw vs. log-transformed) and Normal Q–Q Plots. **(A)** Histogram of the raw CALLY index with kernel density overlay, showing pronounced right-skewness and heavy upper tail. **(B)** Normal Q–Q plot of raw CALLY, indicating substantial departure from normality in the upper quantiles. **(C)** Histogram of ln(CALLY) (natural log), demonstrating improved symmetry and reduced skewness. **(D)** Normal Q–Q plot of ln(CALLY), showing closer alignment to normality with mild residual tail deviations.

In both the NHANES and external validation datasets, the distribution of the original CALLY index exhibited marked right skewness. We therefore (i) generated histograms with kernel density overlays and Q–Q plots for both raw CALLY and ln(CALLY) (Figure 2 for NHANES and Figure 3 for the external cohort), and (ii) conducted pre-specified analyses on both the raw scale (primary) and the ln-transformed scale (sensitivity).

To evaluate the association between the CALLY index and OA prevalence, weighted multivariable logistic regression models were constructed as follows:

- Model 1: Unadjusted;
- Model 2: Adjusted for age, gender, and race/ethnicity;
- Model 3: Further adjusted for smoking status, hypertension, education level, diabetes, and alcohol consumption.

The CALLY index was divided into quartiles, and a trend test across these categories was performed. In parallel, the CALLY index was natural log-transformed (base e) to create ln(CALLY) for regression analyses.

The RCS models were used to assess potential nonlinear associations between the CALLY index and OA prevalence, and smoothed curve fitting was employed to visualize the dose–response association. Effect modification was examined using interaction terms and stratified analyses (such as CALLY × BMI).

To assess external consistency, we examined a de-identified cross-sectional cohort drawn from a general hospital population (n = 1,534; age ≥ 20 years; 505 with OA and 1,029 without OA). Baseline characteristics were summarized according to OA status. Using CALLY quartiles as the main exposure (with Q1 as the reference), we fitted multivariable logistic regression models in three stages (Model 1: unadjusted; Model 2: adjusted for age and sex; Model 3: additionally adjusted for education, smoking, alcohol use, diabetes, and hypertension). Because of the right-skewed distribution of the index, the CALLY index was also transformed using the natural logarithm ln(CALLY) for regression analyses. Nonlinearity and potential inflection points were examined using segmented (two-piece) regression with likelihood ratio tests. Interaction effects (CALLY × age, sex, BMI, diabetes, hypertension) were evaluated using likelihood ratio or Wald *χ*^2^ tests, and subgroup analyses were stratified accordingly.

All statistical analyses were performed using R software (version 4.4.2; R Foundation for Statistical Computing, Vienna, Austria; https://www.r-project.org). A two-sided *p*-value of <0.05 was considered statistically significant.

## 3 Results

### 3.1 Baseline characteristics

A total of 20,315 participants were included in the final analysis, of whom 1,636 (8.1%) self-reported a diagnosis of OA. After applying appropriate sample weights, these figures corresponded to an estimated 4,731,911 adults in the United States population, of whom 2,021,580 (43.0%) reported OA. The baseline characteristics of the study population are summarized in Table 1.

**Table 1:** Baseline characteristics of participants by OA status and CALLY index.

Characteristics	Total (%)	OA No (%)	OA Yes (%)	*p*-value
Un-weighted	20,315 (100)	18,679 (92)	1,636 (8)	<0.001
Weighted		4,731,911	2,021,580	
CALLY index quartile				<0.001
Q1	5,080 (25)	4,482 (24)	598 (37)	
Q2	5,084 (25)	4,619 (25)	465 (28)	
Q3	5,079 (25)	4,734 (25)	345 (21)	
Q4	5,072 (25)	4,844 (26)	228 (16)	
CALLY index (mean ± SD)	11.05 ± 20.17	11.36 ± 20.51	6.89 ± 16.11	<0.001
Age				<0.001
Age ≤60	15,281 (75)	14,579 (78)	702 (43)	
Age >60	5,034 (25)	4,100 (22)	935(57)	
Sex				<0.001
Female	10,043 (49)	9,061 (49)	982 (60)	
Male	10,272 (51)	9,618 (51)	654 (40)	
BMI				<0.001
<25	6,278 (31)	5,903 (32)	375 (23)	
25–30	7,146 (35)	6,677 (36)	469 (29)	
>30	6,891 (34)	6,099 (33)	792 (48)	
Diabetes				<0.001
No	18,292(90)	17,041 (91.2)	1,251 (76)	
Yes	2023(10)	1,638 (8.8)	385 (24)	
Race				<0.001
Mexican American	4,205 (21)	3,973 (21)	232 (14)	
Non-Hispanic White	3,919 (19)	3,525 (19)	394 (24)	
Non-Hispanic Black	9,259 (45)	8,456 (45)	803 (49)	
Other Hispanic	1,651 (8.1)	1,528 (8.2)	123 (7.5)	
Other/multiracial	1,281 (6.3)	1,197(6.1)	84 (5.1)	
Education				<0.001
9–11th Grade	2,929 (14.4)	2,647 (14)	285 (17)	
College Graduate or above	4,558 (22.4)	4,296 (23)	262 (16)	
High School Grad/GED	4,655 (23)	4,286 (23)	369 (23)	
Less than 9th	2,425 (12)	2,173 (12)	252 (15)	
Some College or AA degree	5,745 (28.3)	5,277 (28)	468 (29)	
Smoke				<0.001
No	11,037	10,325 (55)	712 (44)	
Yes	9,278	8,354 (45)	924 (56)	
Drink				<0.001
No	5,829	5,267 (28)	562 (34)	
Yes	14,486	13,142 (72)	1,074 (66)	
HBP				<0.001
No	14,496	13,827 (74)	669 (41)	
Yes	5,819	4,852 (26)	967 (59)	

OA, osteoarthritis; BMI, body mass index; HBP, high blood pressure; CALLY, C-reactive protein–albumin–lymphocyte.

Categorical variables are presented as unweighted counts (*n*) with weighted percentages in parentheses. Continuous variables are presented as weighted means ± standard deviations.

*p*-values were calculated using weighted chi-square tests for categorical variables and weighted linear regression for continuous variables, according to the NHANES analytical guidelines.

The mean CALLY index was significantly lower among participants with OA (6.89 ± 16.11) than among those without OA (11.36 ± 20.51; *p* < 0.001). Quartile distribution analysis further demonstrated that participants with OA were more likely to be in the lowest quartile (Q1) of the CALLY index (37.0%). In contrast, only 14.0% were in the highest quartile (Q4), a statistically significant difference (*p* < 0.001). Compared with non-OA participants, the OA group was older and included higher proportions of women and non-Hispanic Black individuals. Additionally, participants with OA were more likely to have completed high school or an equivalent level of education, to report a history of smoking and alcohol consumption, and to have a diagnosis of hypertension. All group differences were statistically significant (*p* < 0.001).

### 3.2 Distributions

In NHANES, the distribution of the raw CALLY index showed marked right skewness with heavy tails, and after log transformation it appeared more symmetric (Figure 2). The external validation cohort showed a similar distributional pattern (Figure 3).

### 3.3 Association between the CALLY index and OA prevalence

**Figure 4:**
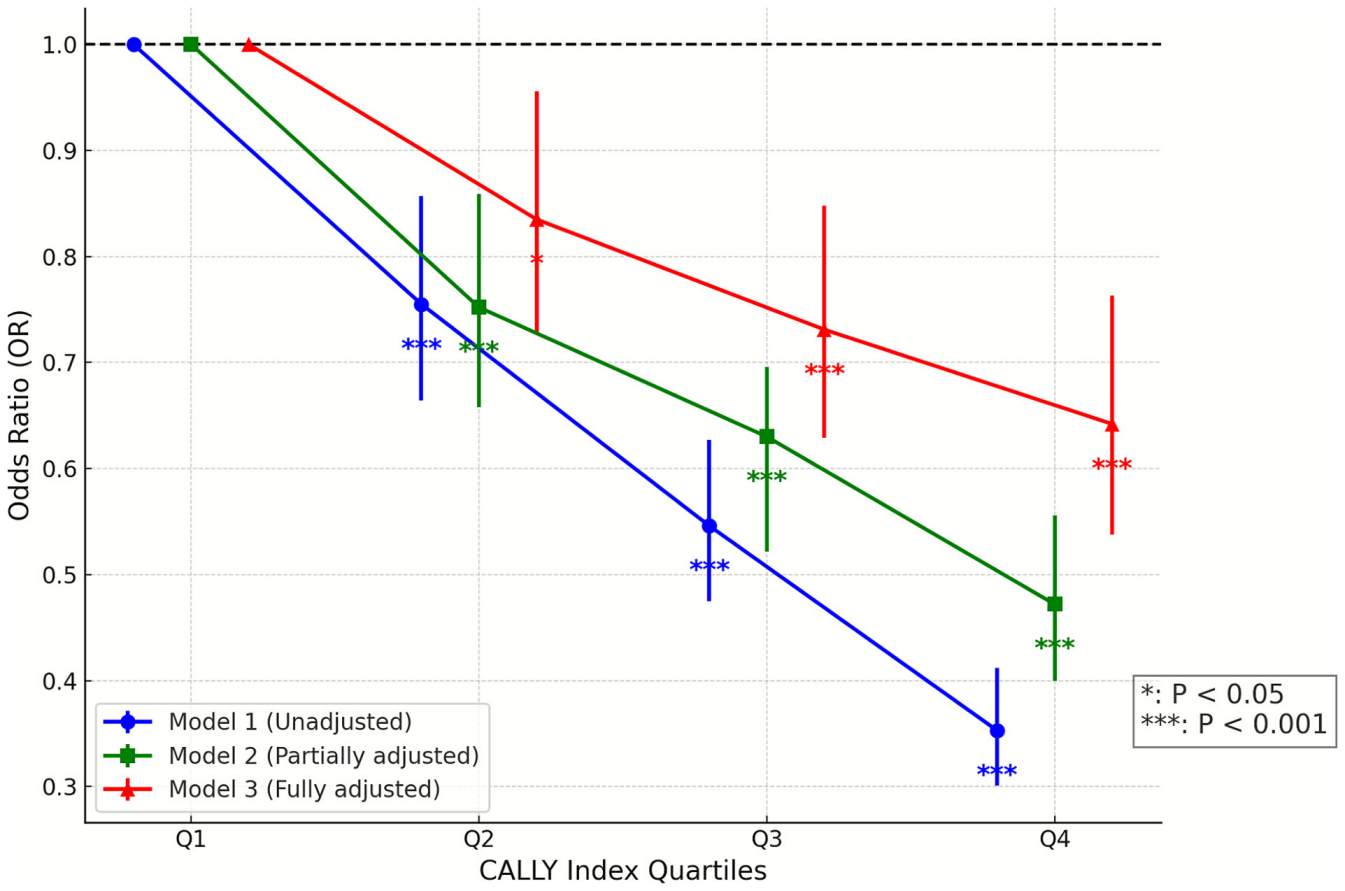
Dose response relationship between CALLY index and OA risk (Model 1–3). Odds ratios (ORs) and 95% confidence intervals (CIs) for OA risk are shown across quartiles (Q1–Q4) of the CALLY index, with Q1 (lowest quartile) used as the reference group. Quartiles were calculated based on the distribution of the CALLY index in the study population. Three models are shown: Model 1 (unadjusted), Model 2 (adjusted for demographic variables: age, sex, race), and Model 3 (fully adjusted for demographic and clinical covariates including BMI, smoking, alcohol, hypertension, diabetes). A clear inverse dose–response relationship is observed, with higher CALLY index quartiles associated with lower OA risk. Error bars represent 95% CIs. Asterisks indicate statistical significance: *p < 0.05, **p < 0.01, ***p < 0.001.

**Figure 5:**
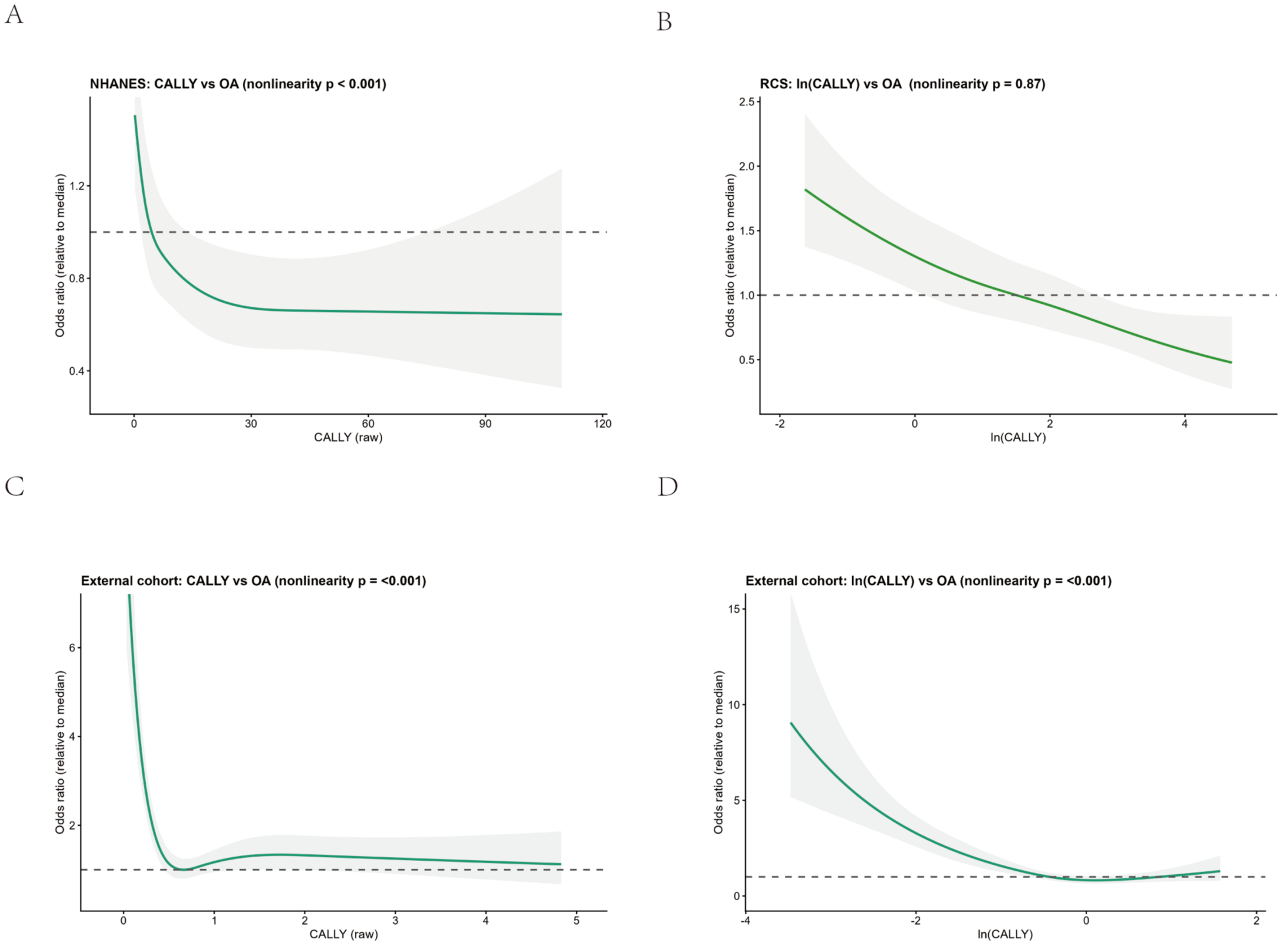
Exposure–response association between the CALLY index and osteoarthritis prevalence: restricted cubic spline (RCS) models in NHANES and an external cohort. **(A)** NHANES: RCS for raw CALLY. **(B)** NHANES: RCS for ln(CALLY) (natural log). **(C)** External cohort: RCS for raw CALLY. **(D)** External cohort: RCS for ln(CALLY).

In the weighted NHANES analyses, both the raw CALLY index and ln(CALLY) were inversely associated with OA prevalence, and the direction of association was consistent across Models 1–3. Although the estimates were modestly attenuated with sequential adjustment, most ORs remained below 1.00 (Table 2), and the log transformation reduced the right-skewness of the CALLY distribution. A graded inverse dose–response pattern was observed (Figure 4). Restricted cubic spline analyses showed evidence of nonlinearity on the raw scale (*p* for non-linearity <0.001) and an approximately log-linear inverse pattern on the log-transformed scale (Figure 5B; *p* for non-linearity = 0.78). At the lower end of the raw CALLY distribution, the odds ratio briefly exceeded 1.0 before decreasing and then flattening around a CALLY value of approximately 4.96, a descriptive curve feature that is not interpreted as a causal threshold. Taken together, these findings support a statistically significant inverse association that is robust to covariate adjustment and consistent across the raw and log-transformed scales. The interpretation is restricted to a cross-sectional framework without predictive or causal claims, and prospective studies are needed to clarify temporal relationships and to examine potential clinical applicability.

**Table 2:** NHANES cohort: multivariable associations of CALLY/ln(CALLY) (per 1−SD increase) with OA prevalence.

Exposure	Model	Effect (OR, 95% CI)	*p*-value
CALLY (per 1 SD)	M1 (unadjusted)	0.59 (0.48, 0.72)	<0.001
	M2 (min-adjusted)	0.75 (0.63, 0.90)	0.002
	M3 (fully-adjusted)	0.78 (0.66, 0.92)	0.005
ln(CALLY) (per 1 SD)	M1 (unadjusted)	0.66 (0.63, 0.70)	<0.001
	M2 (min-adjusted)	0.75 (0.70, 0.80)	<0.001
	M3 (fully-adjusted)	0.77 (0.73, 0.82)	<0.001

OR, odds ratio; CI, confidence interval; SD, standard deviation.

CALLY index: albumin (g/L) × lymphocyte count (10^9/L) ÷ [C-reactive protein (mg/L) × 10]; ln(CALLY) denotes the natural logarithm of the CALLY index.

Models: M1 = unadjusted; M2 = adjusted for age, sex, race/ethnicity, and education; M3 = additionally adjusted for smoking status, alcohol intake, hypertension, and diabetes.

### 3.4 Interaction model regression results

Interaction modeling (Table 3) compared the 25th (1.84) and 75th (11.5) percentiles of the CALLY index and showed that the higher CALLY group had lower odds of OA than the lower CALLY group (OR = 0.504; 95% CI 0.455–0.559). In the corresponding model-based ANOVA (Table 4), the CALLY index was associated with the odds of OA overall (*χ*^2^ = 217, df = 3, *p* < 0.001), and the test for the nonlinear spline terms was also significant (*χ*^2^ = 116, df = 2, *p* < 0.001), consistent with the nonlinearity observed in the RCS analyses. Taken together, these results indicate an inverse, partly nonlinear association between the CALLY index and the odds of OA in the interaction model. The CALLY index may help describe risk strata in observational data, but its potential value for clinical decision-making should be examined in prospective studies.

**Table 3:** Results of interaction model including CALLY index.

Factor	Low	High	OR.95%. CI.	OR Diff
CALLY	1.84	11.5	0.504 (0.455,0.559)	0.104

OR Diff, odds ratio difference.

CALLY index was stratified at the 25th (1.84) and 75th (11.5) percentiles.

The interaction model evaluates the effect of the CALLY index on OA risk at low and high levels, with OR Diff indicating the difference in odds ratios across strata.

**Table 4:** ANOVA results for interaction model including CALLY.

Factor	Chi Square	df	*p*-value
CALLY	217	3	<0.001
Non-linear	116	2	<0.001
TOTAL	217	3	<0.001

Chi-square tests were used to assess the significance of the overall association, the non-linear component, and total model fit in the restricted cubic spline model.

All models were weighted and adjusted for covariates as described in the Methods section.

### 3.5 Subgroup analysis

To assess the consistency of the association between the CALLY index and OA prevalence across populations, we conducted subgroup analyses stratified by age, sex, BMI, hypertension, and diabetes. As shown in Table 5, higher CALLY levels were generally inversely associated with OA prevalence across most subgroups. In certain higher-risk strata—such as older adults and individuals with diabetes—the magnitude and statistical significance of the association were attenuated, suggesting potential effect modification.

**Table 5:** Stratified analysis of the association between CALLY index and OA risk.

Subgroup	CALLY index quartile (Q1–Q4)	OR	CI	*p*-value
Age ≤60	Q1	ref	ref	
	Q2	0.844	0.680–1.047	<0.05
	Q3	0.731	0.575–0.926	<0.01
	Q4	0.542	0.411–0.711	<0.001
Age >60	Q1	ref	ref	
	Q2	0.876	0.728–1.053	<0.05
	Q3	0.773	0.631–0.945	<0.01
	Q4	0.815	0.640–1.033	<0.001
Male	Q1	ref	ref	
	Q2	0.747	0.603–0.926	<0.01
	Q3	0.639	0.509–0.802	<0.001
	Q4	0.667	0.515–0.863	<0.01
Female	Q1	ref	ref	
	Q2	0.900	0.754–1.075	0.247
	Q3	0.814	0.665–0.997	<0.05
	Q4	0.716	0.563–0.912	<0.01
BMI < 25	Q1	ref	ref	
	Q2	0.869	0.628–1.202	0.395
	Q3	0.625	0.455–0.860	0.004
	Q4	0.471	0.346–0.644	<0.001
BMI ≥ 25	Q1	ref	ref	
	Q2	0.909	0.780–1.059	0.222
	Q3	0.807	0.677–0.959	0.016
	Q4	0.660	0.526–0.823	<0.001
HBP Yes	Q1	ref	ref	
	Q2	0.870	0.723–1.047	0.140
	Q3	0.791	0.644–0.973	0.026
	Q4	0.734	0.570–0.945	0.017
HBP No	Q1	ref	ref	
	Q2	0.903	0.727–1.122	0.357
	Q3	0.768	0.607–0.971	0.028
	Q4	0.754	0.580–0.979	0.034
Diabetes Yes	Q1	ref	ref	
	Q2	0.856	0.634–1.156	0.309
	Q3	0.693	0.490–0.980	0.038
	Q4	0.831	0.553–1.249	0.372
Diabetes No	Q1	ref	ref	
	Q2	0.891	0.759–1.045	0.155
	Q3	0.809	0.680–0.962	0.016
	Q4	0.736	0.601–0.901	0.003

Age, sex, BMI, HBP, and diabetes status were used in stratified logistic regression models. Quartiles of the CALLY index (Q1–Q4) were used, and models were adjusted for demographic and clinical covariates within each subgroup.

### 3.6 External validation findings

Baseline characteristics of the external cohort are summarized in Table 6. Compared with the non-OA group, participants with OA were older, more likely to be female, more likely to smoke, and more likely to have diabetes or hypertension, and they had significantly lower CALLY index values (all *p* < 0.001). Quartile distribution indicated that participants with OA were predominantly in Q1, whereas non-OA participants were mainly in Q3–Q4 (*p* < 0.001).

**Table 6:** Baseline characteristics of participants by OA status and CALLY index (external validation cohort).

Characteristics	OA status			*p*-value
	No *N* = 1,029	Yes *N* = 505	Overall *N* = 1,534	
CALLY index				<0.001
Mean ± SD	11.62 ± 41.15	6.66 ± 7.58	9.99 ± 34.05	
CALLY quartile				<0.001
Q1	153 (14.9%)	230 (45.5%)	383 (25.0%)	
Q2	289 (28.1%)	94 (18.6%)	383 (25.0%)	
Q3	298 (29.0%)	86 (17.0%)	384 (25.0%)	
Q4	289 (28.1%)	95 (18.8%)	384 (25.0%)	
Age group				<0.001
≤60	667 (64.8%)	175 (34.7%)	842 (54.9%)	
>60	362 (35.2%)	330 (65.3%)	692 (45.1%)	
Sex				<0.001
Female	456 (44.3%)	335 (66.3%)	791 (51.6%)	
Male	573 (55.7%)	170 (33.7%)	743 (48.4%)	
BMI category				<0.001
<25	339 (32.9%)	190 (37.6%)	529 (34.5%)	
25–30	361 (35.1%)	236 (46.7%)	597 (38.9%)	
>30	329 (32.0%)	79 (15.6%)	408 (26.6%)	
Diabetes	121 (11.8%)	108 (21.4%)	229 (14.9%)	<0.001
Education				0.004
Less than 9th grade	142 (13.8%)	87 (17.2%)	229 (14.9%)	
9–11th grade	242 (23.5%)	79 (15.6%)	321 (20.9%)	
High school grad/GED or equivalent	232 (22.5%)	118 (23.4%)	350 (22.8%)	
Some college or AA degree	126 (12.2%)	78 (15.4%)	204 (13.3%)	
College graduate or above	287 (27.9%)	143 (28.3%)	430 (28.0%)	
Smoke	65 (6.3%)	66 (13.1%)	131 (8.5%)	<0.001
Drink	100 (9.7%)	76 (15.0%)	176 (11.5%)	0.003
Hypertension	350 (34.0%)	276 (54.7%)	626 (40.8%)	<0.001

OA status: yes/no (self-reported physician diagnosis of OA).

CALLY quartile: Q1–Q4 in ascending order of CALLY.

Age group: ≤60 years vs. >60 years.

Sex: female/male.

BMI category: <25, 25–30, >30 kg/m^2.

Diabetes, Smoke, Drink, Hypertension (HBP): yes/no.

Education (1–5): 1 = Less than 9th grade; 2 = 9–11th grade; 3 = High school graduate/GED or equivalent; 4 = Some college or AA degree; 5 = College graduate or above.

To synthesize the findings from the external consistency analysis, we presented the multivariable regression results in a concise three-line table (Table 7). In the independent hospital-based cohort, each 1-standard-deviation (1−SD) increase in ln(CALLY) was associated with lower odds of OA across all model specifications (Models 1–3), and the adjusted odds ratios remained consistently below 1.00. When the raw CALLY index (per 1−SD increase) was used as the exposure, the direction of association was similar, although the magnitude was slightly attenuated in the fully adjusted model. These findings were directionally consistent with those in the main NHANES cohort and support reporting results on both the raw and log-transformed scales: log transformation reduces right skewness and yields an approximately linear pattern in dose–response visualization, whereas the raw scale retains the nonlinearity observed in the primary analyses. All estimates are reported within a cross-sectional framework and do not imply causal inference. Based on the interaction-adjusted model (Table 8), participants at the 75th percentile (CALLY ≈ 10.67) had an OR of 0.64 (95% CI 0.49–0.84) compared with those at the 25th percentile (≈ 3.47). ANOVA results (Table 9) indicated significant overall and nonlinear components of the association (*χ*^2^-total = 101–303, *p* < 0.001), which were consistent in direction and magnitude with the main NHANES results (Q4 vs. Q1, fully adjusted OR ≈ 0.64).

**Table 7:** Association between CALLY quartiles and osteoarthritis (OA) across models (external validation).

Exposure	Model	Effect (OR, 95% CI)	*P*-value
CALLY (per 1 SD)	M1 (unadjusted)	0.19 (0.10, 0.36)	<0.001
	M2 (min-adjusted)	0.26 (0.14, 0.48)	<0.001
	M3 (fully-adjusted)	0.25 (0.14, 0.47)	<0.001
ln(CALLY) (per 1 SD)	M1 (unadjusted)	0.54 (0.48, 0.60)	<0.001
	M2 (min-adjusted)	0.57 (0.51, 0.65)	<0.001
	M3 (fully-adjusted)	0.56 (0.50, 0.64)	<0.001

Models: M1 = unadjusted; M2 = adjusted for age, sex, race/ethnicity, and education; M3 = additionally adjusted for smoking status, alcohol intake, hypertension, and diabetes.

**Table 8:** Results of the interaction model, including the CALLY index (external validation).

Factor	Low	High	OR.95%. CI.	OR Diff
CALLY^†^	3.47	10.67	0.642 (0.494–0.835)	0.341

Definition of “Low/High”: 25th percentile (Low = 3.47) and 75th percentile (High = 10.67) of the CALLY distribution in the external cohort.

Model: Logistic regression modeling CALLY as a continuous predictor using restricted cubic splines; ORs are reported for High vs. Low with covariates fixed at their reference/mean (same adjustment set as Model 3 in Table 9).

OR Diff: absolute difference in ORs between the two percentiles.

**Table 9:** ANOVA results for the interaction model, including CALLY (external validation).

Factor	Chi Square	df	*p*-value
CALLY	101	3	<0.001
Non-linear	86	2	<0.001
TOTAL	303	14	<0.001

Components:

• CALLY = overall contribution of the CALLY term(s).

• Nonlinear = contribution of spline (nonlinear) components beyond the linear term.

• TOTAL = overall model χ^2.

Model and adjustment: same interaction/spline logistic model as in Table 10, adjusted for age, sex, education, BMI, smoking, drinking, diabetes, and HBP.

*p*-values: likelihood-ratio χ^2 tests (two-sided).

In the RCS analysis, the association between CALLY and OA (Figure 5) was non-linear (*p* < 0.001), with the curve plateauing at a CALLY value of approximately 4.96. This pattern is considered exploratory and suggests a possible plateau or saturation pattern in the association at higher CALLY levels. In contrast, ln(CALLY) showed an approximately linear inverse association with OA (test for nonlinearity *p* > 0.05). Using the median of ln(CALLY) as the reference, the odds ratio for OA decreased progressively as ln(CALLY) increased. Subgroup analyses (Table 10) were generally consistent with the NHANES findings, showing inverse associations across multiple groups; the associations were weaker in the older and diabetic subgroups.

**Table 10:** Stratified analysis of the association between CALLY quartiles and OA risk (external validation).

Subgroup	OR by CALLY quartiles (Q1 ref)	OR	CI	*p*-value
Age ≤60	Q1	ref	ref	ref
	Q2	0.177	0.105–0.293	<0.001
	Q3	0.172	0.101–0.287	<0.001
	Q4	0.237	0.144–0.384	<0.001
Age >60	Q1	ref	ref	ref
	Q2	0.307	0.188–0.496	<0.001
	Q3	0.233	0.141–0.380	<0.001
	Q4	0.252	0.152–0.411	<0.001
Male	Q1	ref	ref	ref
	Q2	0.220	0.118–0.400	<0.001
	Q3	0.213	0.116–0.383	<0.001
	Q4	0.300	0.169–0.526	<0.001
Female	Q1	ref	ref	ref
	Q2	0.252	0.159–0.394	<0.001
	Q3	0.181	0.112–0.288	<0.001
	Q4	0.228	0.141–0.365	<0.001
BMI < 25	Q1	ref	ref	ref
	Q2	0.178	0.095–0.327	<0.001
	Q3	0.143	0.074–0.269	<0.001
	Q4	0.257	0.141–0.461	<0.001
BMI ≥ 25	Q1	ref	ref	ref
	Q2	0.273	0.178–0.414	<0.001
	Q3	0.255	0.165–0.388	<0.001
	Q4	0.259	0.165–0.401	<0.001
HBP Yes	Q1	ref	ref	ref
	Q2	0.167	0.096–0.282	<0.001
	Q3	0.213	0.125–0.357	<0.001
	Q4	0.274	0.165–0.449	<0.001
HBP No	Q1	ref	ref	ref
	Q2	0.335	0.211–0.528	<0.001
	Q3	0.222	0.134–0.361	<0.001
	Q4	0.246	0.147–0.405	<0.001
Diabetes Yes	Q1	ref	ref	ref
	Q2	0.182	0.056–0.549	0.003
	Q3	0.086	0.027–0.253	<0.001
	Q4	0.175	0.052–0.546	0.003
Diabetes No	Q1	ref	ref	ref
	Q2	0.257	0.177–0.369	<0.001
	Q3	0.235	0.160–0.342	<0.001
	Q4	0.268	0.185–0.387	<0.001

Model: Within each stratum, logistic regression with CALLY quartiles (Q1 reference).

Adjustment: Each stratum is adjusted for the full covariate set used in Table 9 Model 3—age, sex, education, BMI, smoking, drinking, diabetes, and HBP—excluding the stratifying variable itself to avoid collinearity.

*p*-values: Wald tests for the quartile indicators within each stratum.

Strata definitions: Age (≤60 vs. >60 years); Sex (male/female); BMI (<25 vs. ≥25 kg/m^2); HBP (yes/no); Diabetes (yes/no).

## 4 Discussion

Using NHANES data from 2001 to 2010 and 2015–2018 (*n* = 20,315), this cross-sectional analysis identified an inverse association between the CALLY index and OA prevalence after adjusting for multiple covariates. On the raw scale, RCS modeling indicated a steep inverse association at lower CALLY values, which then gradually plateaued in the mid- to high range. After log transformation to ln(CALLY), the association was approximately linear.

The CALLY index is a composite marker that integrates information on nutritional status, immune function, and systemic inflammation, calculated from serum albumin levels, lymphocyte counts, and CRP levels (25–27). Given that nutritional, immunological, and inflammatory factors are important in OA pathogenesis (28, 29), we interpreted the CALLY index–OA association in the context of these three domains.

Traditionally, OA was viewed as a mechanical wear-and-tear condition. However, increasing evidence suggests that OA is a systemic disorder that is influenced by biological aging, chronic inflammation, and metabolic dysfunction (30). Nutritional status, particularly in older adults, has been strongly linked to the occurrence and progression of chronic diseases. Serum albumin is a well-established biomarker of nutritional status and an indicator of the severity of chronic illness. Individuals with OA frequently exhibit hypoalbuminemia, which has been associated with increased disease severity and poorer quality of life. Mechanistically, nutritional deficiencies may impair cartilage matrix synthesis and reduce anti-inflammatory capacity, which may contribute to more rapid joint degeneration (31, 32). Consistent with these findings, we observed that lower CALLY index values—driven in part by low serum albumin—were statistically associated with higher OA prevalence.

Although OA is not classically defined as an immune-mediated disorder, accumulating evidence supports the involvement of immune dysregulation, particularly lymphocytes, in OA pathogenesis (33). As the lymphocyte component of the CALLY index reflects immune competence, its reduction may indicate immunosenescence, a common feature in older adults. This condition impairs the resolution of inflammation, potentially leading to persistent synovitis and the activation of inflammatory signaling pathways, such as NF-κB, mTOR, and JAK/STAT (34). Our findings align with this perspective: higher CALLY index values, reflective of greater lymphocyte counts, were statistically associated with a lower prevalence of OA.

Inflammation represents a third key dimension of the CALLY index. Pro-inflammatory cytokines such as IL-1β, IL-6, TNF, and oncostatin M promote cartilage catabolism by suppressing type II collagen and aggrecan synthesis (35). Activated macrophages (such as CD68^+^ cells) may exacerbate joint damage by phagocytosing collagen fragments and presenting them to CD4^+^ T cells, thereby perpetuating immune-mediated cartilage degradation (36). Moreover, systemic inflammation can induce muscle atrophy (37) and disrupt metabolic function, which may contribute to the development and progression of OA (38). In our analysis, elevated CRP—reflected in a lower CALLY index—was significantly associated with increased OA prevalence, in line with the pro-inflammatory hypothesis of OA pathophysiology.

Taken together, our findings highlight the interrelated roles of nutrition, immunity, and inflammation in the development of OA. These factors likely interact in a self-reinforcing cycle, where malnutrition impairs cartilage repair and reduces muscle mass, immune senescence compromises inflammatory control, and chronic inflammation exacerbates nutrient depletion (39, 40). The CALLY index, as a composite marker, summarizes these intertwined processes and may be informative for risk stratification in OA within observational settings.

To better understand statistical associations rather than causal pathways, we performed interaction-term testing with stratified analyses. The results from interaction and stratified tests provided evidence of a nonlinear inverse association across multiple subgroups (by age, BMI, diabetes, and hypertension), with an overall consistent direction.

In an independent cohort, we observed a consistent nonlinear inverse association, providing external agreement with the NHANES findings and suggesting that the CALLY index may be informative for risk stratification in observational settings. In both datasets, the dose–response curves exhibited a similar pattern: OA prevalence was lower at higher CALLY values, with a relatively steep inverse association at lower CALLY values that then plateaued in the mid-to-high range. Fully adjusted effect estimates were highly consistent across cohorts (external OR ≈ 0.64; NHANES Q4 vs. Q1 OR ≈ 0.64), which supports the internal consistency and robustness of the observed associations.

Notably, a recent study by Geng and Zhang (41) was the first to examine the association between the CALLY index and OA using NHANES data. Their analysis demonstrated a significant nonlinear inverse association and proposed a classification model for OA status (area under the curve [AUC] = 0.825). Compared with their work, our study extends the literature in several important ways: (1) we incorporated the most recent NHANES cycles through 2018, improving temporal representativeness; (2) we applied a log transformation to the raw CALLY index to mitigate right skew and enhance statistical stability; (3) we performed subgroup interaction analyses to evaluate the consistency of the association across different population strata; and (4) we assessed external consistency in an independent cohort. These extensions not only broaden the generalizability of the findings and deepen mechanistic insight, but also—despite differences in effect size—demonstrate a directionally consistent association, thereby supporting the robustness of the evidence base.

This study has several strengths. It uses nationally representative NHANES data along with an independent external validation cohort; employs flexible yet understandable modeling techniques (survey-weighted logistic regression and restricted cubic spline analyses on the raw CALLY scale), with a prespecified sensitivity analysis using ln(CALLY); and shows a consistently inverse association across analytic approaches. To lessen the risk of overadjustment, the primary models intentionally avoided conditioning on BMI. All conclusions are framed in terms of statistical associations rather than causal effects.

Although this study has strengths, it also faces several limitations. The cross-sectional design prevents establishing temporality or drawing causal relationships. The lack of direct body-composition measures (such as DXA) hinders the ability to distinguish between the contributions of lean and fat mass within BMI. Measurement error and residual confounding may exist in the CALLY components. Additionally, differences between cohorts in sampling and weighting strategies, laboratory platforms, covariate availability, and single-time-point measurements may contribute to heterogeneity in effect sizes.

Considering the NHANES and external-cohort results together, we observed consistent directional agreement. However, due to differences between cohorts in sampling and weighting, laboratory platforms, and covariate availability, effect sizes should not be directly compared, and clinical thresholds or intervention implications should not be inferred. Overall, the findings suggest a consistent inverse association between CALLY and OA prevalence; however, the causal mechanisms and clinical implications remain uncertain and warrant evaluation in prospective studies with temporal data and direct body-composition measures, such as DXA.

## 5 Conclusion

Using NHANES data, with consistency evaluated in an independent external cohort, we observed a strong inverse association between the CALLY index and OA prevalence; the association was nonlinear on the raw scale (with a sharper change at lower CALLY levels) and approximately linear on the ln(CALLY) scale. The direction of the association remained consistent across multivariable adjustments and log-transformed sensitivity analyses. Because of the cross-sectional design and the lack of direct body-composition measurements, we do not draw causal inferences; questions of temporality and clinical implications require further investigation in prospective studies with temporal information and body-composition assessments (such as DXA).

## Data Availability

The original contributions presented in the study are included in the article/Supplementary material, further inquiries can be directed to the corresponding author.

## References

[1] Courties A Kouki I Soliman N Mathieu S Sellam J. Osteoarthritis year in review 2024: epidemiology and therapy. Osteoarthr Cartil. (2024) 32:1397–404. doi: 10.1016/j.joca.2024.07.014, 39103081

[2] Wu R Guo Y Chen Y Zhang J. Osteoarthritis burden and inequality from 1990 to 2021: a systematic analysis for the global burden of disease study 2021. Sci Rep. (2025) 15:8305. doi: 10.1038/s41598-025-93124-z, 40065123 PMC11894191

[3] Qiao L Li M Deng F Wen X Wang J Deng H . Epidemiological trends of osteoarthritis at the global, regional, and national levels from 1990 to 2021, with a projection from 2021 to 2050. (2024). doi: 10.1186/s13075-025-03658-w PMC12570834 41152934

[4] Ya-xian D Yan Y Jun Z Qiujun Z Hongyu W. Evidence on risk factors for knee osteoarthritis in middle-older aged: a systematic review and meta analysis. J Orthop Surg Res. (2023) 18:634. doi: 10.1186/s13018-023-04089-6 37641050 PMC10464102

[5] Guizheng W Ke L Muhammad U Zhenglin Z William WL John RS . Risk of metabolic abnormalities in osteoarthritis: a new perspective to understand its pathological mechanisms. Bone Res. (2023) 11:63. doi: 10.1038/s41413-023-00301-9 38052778 PMC10698167

[6] Nedunchezhiyan U Varughese I Sun AR Wu X Crawford R Prasadam I. Obesity, inflammation, and immune system in osteoarthritis. Front Immunol. (2022) 13:907750. doi: 10.3389/fimmu.2022.907750, 35860250 PMC9289681

[7] He Y Li Z Alexander PG Ocasio-Nieves BD Yocum L Lin H . Pathogenesis of osteoarthritis: risk factors, regulatory pathways in chondrocytes, and experimental models. Biology. (2020) 9:194. doi: 10.3390/biology9080194, 32751156 PMC7464998

[8] Zhai G Huang J. Genetics of osteoarthritis. Best Pract Res Clin Rheumatol. (2024) 38:101972. doi: 10.1016/j.berh.2024.101972, 38971692

[9] Allen KD Thoma L Golightly Y. Epidemiology of osteoarthritis. Osteoarthr Cartil. (2021) 30:184–95. doi: 10.1016/j.joca.2021.04.020, 34534661 PMC10735233

[10] Wang S Li W Zhang P Wang Z Ma X Liu C . Mechanical overloading induces GPX4-regulated chondrocyte ferroptosis in osteoarthritis via Piezo1 channel facilitated calcium influx. J Adv Res. (2022) 41:63–75. doi: 10.1016/j.jare.2022.01.004, 36328754 PMC9637484

[11] Liu Y Chen P Hu B Xiao Y Su T Luo X . Excessive mechanical loading promotes osteoarthritis development by upregulating Rcn2. Biochim Biophys Acta Mol basis Dis. (2024) 1870:167251. doi: 10.1016/j.bbadis.2024.167251, 38795835

[12] Pettenuzzo S Berardo A Belluzzi E Pozzuoli A Ruggieri P Carniel EL . Mechanical insights into fat pads: a comparative study of infrapatellar and suprapatellar fat pads in osteoarthritis. Connect Tissue Res. (2025) 66:272–83. doi: 10.1080/03008207.2025.2502591, 40340764

[13] Schulze-Tanzil G. Intraarticular ligament degeneration is interrelated with cartilage and bone destruction in osteoarthritis. Cells. (2019) 8:990. doi: 10.3390/cells8090990, 31462003 PMC6769780

[14] Melrose J. The importance of the knee joint meniscal fibrocartilages as stabilizing weight bearing structures providing global protection to human knee-joint tissues. Cells. (2019) 8:324. doi: 10.3390/cells8040324, 30959928 PMC6523218

[15] Varela-Eirín M Carpintero-Fernández P Guitián-Caamaño A Varela-Vázquez A García-Yuste A Sánchez-Temprano A . Extracellular vesicles enriched in connexin 43 promote a senescent phenotype in bone and synovial cells contributing to osteoarthritis progression. Cell Death Dis. (2022) 13:681. doi: 10.1038/s41419-022-05089-w, 35931686 PMC9355945

[16] Matthew CB Sheth K Rong L Lu D PvK E Archana B . Increased risk of osteoarthritis in patients with atopic disease. Ann Rheum Dis. (2023) 82:866–72. doi: 10.1136/ard-2022-223640 36987654 PMC10314085

[17] Berenbaum F Wallace I Lieberman D Felson D. Modern-day environmental factors in the pathogenesis of osteoarthritis. Nat Rev Rheumatol. (2018) 14:674–81. doi: 10.1038/s41584-018-0073-x, 30209413

[18] Akul M Santul B Hubbard D Hunter M Kolhe R Fulzele S. Advances in molecular biomarker for early diagnosis of osteoarthritis. Biomol Concepts. (2019) 10:111–9. doi: 10.1515/bmc-2019-0014 31401621

[19] Iida H Tani M Komeda K Nomi T Matsushima H Tanaka S . Superiority of CRP-albumin-lymphocyte index (CALLY index) as a non-invasive prognostic biomarker after hepatectomy for hepatocellular carcinoma. HPB. (2022) 24:101–15. doi: 10.1016/j.hpb.2021.06.414, 34244053

[20] Yang M Lin S-Q Liu X-Y Tang M Hu C-L Wang Z-W . Association between C-reactive protein-albumin-lymphocyte (CALLY) index and overall survival in patients with colorectal cancer: from the investigation on nutrition status and clinical outcome of common cancers study. Front Immunol. (2023) 14:14. doi: 10.3389/fimmu.2023.1131496, 37063910 PMC10098202

[21] Xu Z Tang J Xin C Jin Y Zhang H Liang R. Associations of C-reactive protein-albumin-lymphocyte (CALLY) index with cardiorenal syndrome: insights from a population-based study. Heliyon. (2024) 10:e37197. doi: 10.1016/j.heliyon.2024.e37197, 39296012 PMC11408039

[22] Wang Q-W Man G Choi BC-y Yeung Y-m Qiu J Lu X-m . The predictors to self-reported and performance-based physical function in knee osteoarthritis patients: a cross-sectional study. Front Cell Dev Biol. (2024) 12:1406830. doi: 10.3389/fcell.2024.1406830 38946798 PMC11214303

[23] Xu Y Wu Q. Trends and disparities in osteoarthritis prevalence among US adults, 2005–2018. Sci Rep. (2021) 11:21845. doi: 10.1038/s41598-021-01339-7, 34750468 PMC8576014

[24] Yingjun L Jiahao Z Jiayao F Cai S Fan C Zhong Y . Associations of urinary levels of phenols and parabens with osteoarthritis among US adults in NHANES 2005-2014. Ecotoxicol Environ Saf. (2020) 192:110293. doi: 10.1016/j.ecoenv.2020.110293 32045785

[25] Yu D Yuxia L Jianjian Y Cheng-Sen C Lina F Jie Z . The association between the CALLY index and all-cause mortality in patients with COPD: results from the cohort study of NHANES 2007–2010. Int J Chron Obstruct Pulmon Dis. (2025) 20:159–69. doi: 10.2147/COPD.S485036 39867991 PMC11766151

[26] Fukushima N Takahiro M Tsuboi K Keita T Yuda M Fujisaki M . Prognostic significance of the preoperative C-reactive protein-albumin-lymphocyte (CALLY) index on outcomes after gastrectomy for gastric cancer. Surg Today. (2024) 54:943–52. doi: 10.1007/s00595-024-02813-1 38491233

[27] Müller L Hahn F Mähringer-Kunz A Stoehr F Gairing SJ Michel M . Immunonutritive scoring for patients with hepatocellular carcinoma undergoing Transarterial chemoembolization: evaluation of the CALLY index. Cancer. (2021) 13:5018. doi: 10.3390/cancers13195018, 34638502 PMC8508385

[28] Wei N Dai Z. The role of nutrition in osteoarthritis: a literature review. Clin Geriatr Med. (2022) 38:303–22. doi: 10.1016/j.cger.2021.11.006, 35410682

[29] Motta F Barone E Sica A Selmi C. Inflammaging and osteoarthritis. Clin Rev Allergy Immunol. (2023) 64:222–38. doi: 10.1007/s12016-022-08941-1, 35716253

[30] Greene M Greene M Loeser R. Aging-related inflammation in osteoarthritis. Osteoarthr Cartil. (2015) 23:1966–71. doi: 10.1016/j.joca.2015.01.008, 26521742 PMC4630808

[31] Kojima N Kim M Saito K Yoshida Y Hirano H Obuchi S . Predictors of self-reported knee osteoarthritis in community-dwelling older women in Japan: a cross-sectional and longitudinal cohort study. Arch Gerontol Geriatr. (2017) 73:125–32. doi: 10.1016/j.archger.2017.07.005, 28802215

[32] Maheshwari V Choudhury AK Yadav R Dhingra M Kant R Kalia RB. Prevalence of poor nutrition in knee osteoarthritis patients: a hospital-based cohort study in Indian population. Indian J Orthop. (2024) 58:298–307. doi: 10.1007/s43465-023-01090-3, 38425822 PMC10899134

[33] De Roover A Escribano-Núñez A Monteagudo S Lories R. Fundamentals of osteoarthritis: inflammatory mediators in osteoarthritis. Osteoarthr Cartil. (2023) 31:1303–11. doi: 10.1016/j.joca.2023.06.005, 37353140

[34] Shaoshan L Guifeng Z Nan L Zheng W Liaodong L. The interplay of aging and PANoptosis in osteoarthritis pathogenesis: implications for novel therapeutic strategies. J Inflamm Res. (2025) 18:1951–67. doi: 10.2147/JIR.S489613 39959642 PMC11829118

[35] Fahy N Melle MDV Lehmann J Wei W Grotenhuis N Farrell E . Human osteoarthritic synovium impacts chondrogenic differentiation of mesenchymal stem cells via macrophage polarisation state. Osteoarthr Cartil. (2014) 22:1167–75. doi: 10.1016/j.joca.2014.05.021 24911520

[36] Saito I Koshino T Nakashima K Uesugi M Saito T. Increased cellular infiltrate in inflammatory synovia of osteoarthritic knees. Osteoarthr Cartil. (2002) 10:156–62. doi: 10.1053/joca.2001.0494, 11869075

[37] Sebastiaan D Koppo K. Is inflammatory signaling involved in disease-related muscle wasting? Evidence from osteoarthritis, chronic obstructive pulmonary disease and type II diabetes. Exp Gerontol. (2020) 137:110964. doi: 10.1016/j.exger.2020.110964 32407865

[38] Meng Y Han P Ma X He Y Chen H Ren H. Research progress on the mechanism of acute hypertriglyceridemic pancreatitis. Pancreas. (2024) 53:e700–9. doi: 10.1097/MPA.0000000000002364, 38696438

[39] Nidhi K-M David KF. Competition for nutrients and its role in controlling immune responses. Nat Commun. (2019) 10. doi: 10.1038/s41467-019-10015-4 PMC6509329 31073180

[40] Arie U Rahardjo E Perdanakusuma DS. Effects of albumin infusion on serum levels of albumin, proinflammatory cytokines (TNF-α, IL-1, and IL-6), CRP, and MMP-8; tissue expression of EGRF, ERK1, ERK2, TGF-β, collagen, and MMP-8; and wound healing in Sprague Dawley rats. Int J Inflamm. (2020) 2020:1–13. doi: 10.1155/2020/3254017 PMC7256723 32518615

[41] Geng M Zhang K. CRP-albumin-lymphocyte index (CALLYI) as a risk-predicting biomarker in association with osteoarthritis. Arthritis Res Ther. (2025) 27:57. doi: 10.1186/s13075-025-03530-x, 40108660 PMC11921634

